# Ethyl 1-phenyl-2-[4-(trifluoro­meth­yl)phen­yl]-1*H*-benzimidazole-5-carboxyl­ate

**DOI:** 10.1107/S1600536812022210

**Published:** 2012-05-23

**Authors:** Yeong Keng Yoon, Mohamed Ashraf Ali, Tan Soo Choon, Suhana Arshad, Ibrahim Abdul Razak

**Affiliations:** a Institute for Research in Molecular Medicine, Universiti Sains Malaysia, Minden 11800, Penang, Malaysia; b School of Physics, Universiti Sains Malaysia, 11800 USM, Penang, Malaysia

## Abstract

The asymmetric unit of the title compound, C_23_H_17_F_3_N_2_O_2_, contains two mol­ecules. In one of the mol­ecules, the phenyl and triflouromethyl-substituted benzene rings form dihedral angles of 52.05 (8) and 33.70 (8)°, respectively, with the benzimidazole ring system, while the dihedral angle between them is 58.24 (10)°. The corresponding values in the other mol­ecule are 58.40 (8), 25.90 (8) and 60.83 (10)°, respectively. In the crystal, mol­ecules are linked into chains along [100] by C—H⋯O and C—H⋯N hydrogen bonds. Aromatic π–π stacking inter­actions [centroid–centroid distance = 3.6700 (12) Å] also occur.

## Related literature

For background to benzimidazole derivatives as drugs, see: Spasov *et al.* (1999 ▶); Grassmann *et al.* (2002 ▶); Demirayak *et al.* (2002 ▶); Evans *et al.* (1997 ▶). For related structures, see: Yoon *et al.* (2011 ▶); Kassim *et al.* (2012 ▶). For stability of the temperature controller used in the data collection, see: Cosier & Glazer (1986 ▶).
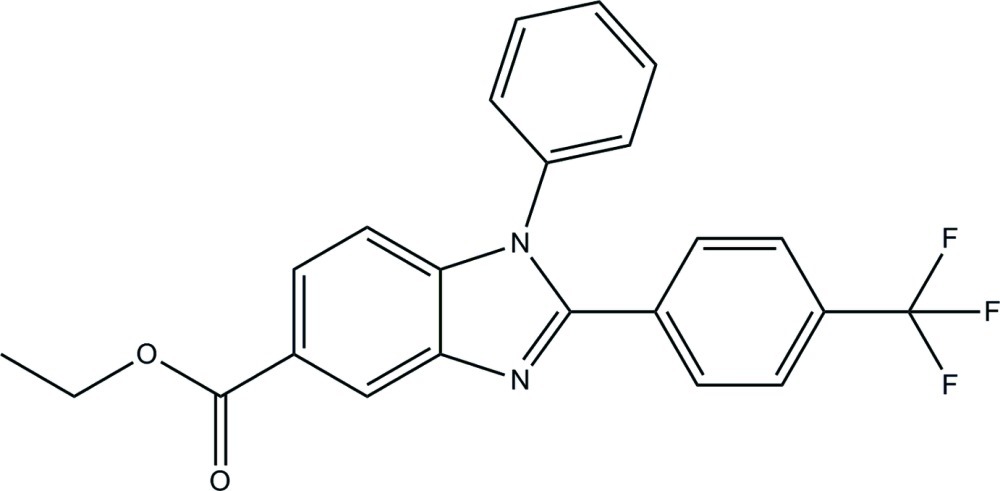


## Experimental

### 

#### Crystal data

C_23_H_17_F_3_N_2_O_2_
*M* *_r_* = 410.39
Monoclinic, 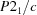

*a* = 9.8548 (2) Å
*b* = 25.0714 (6) Å
*c* = 16.0566 (4) Å
β = 107.023 (1)°
*V* = 3793.35 (15) Å^3^
*Z* = 8
Mo *K*α radiation
μ = 0.11 mm^−1^
*T* = 100 K
0.43 × 0.37 × 0.28 mm

#### Data collection

Bruker SMART APEXII CCD diffractometer
Absorption correction: multi-scan (*SADABS*; Bruker, 2009 ▶) *T*_min_ = 0.953, *T*_max_ = 0.969
36855 measured reflections
10921 independent reflections
5999 reflections with *I* > 2σ(*I*)
*R*_int_ = 0.052

#### Refinement

*R*[*F* ^2^ > 2σ(*F* ^2^)] = 0.062
*wR*(*F* ^2^) = 0.142
*S* = 1.03
10921 reflections
543 parameters
H-atom parameters constrained
Δρ_max_ = 0.30 e Å^−3^
Δρ_min_ = −0.35 e Å^−3^

### 

Data collection: *APEX2* (Bruker, 2009 ▶); cell refinement: *SAINT* (Bruker, 2009 ▶); data reduction: *SAINT*; program(s) used to solve structure: *SHELXTL* (Sheldrick, 2008 ▶); program(s) used to refine structure: *SHELXTL*; molecular graphics: *SHELXTL*; software used to prepare material for publication: *SHELXTL* and *PLATON* (Spek, 2009 ▶).

## Supplementary Material

Crystal structure: contains datablock(s) global, I. DOI: 10.1107/S1600536812022210/hb6782sup1.cif

Structure factors: contains datablock(s) I. DOI: 10.1107/S1600536812022210/hb6782Isup2.hkl

Supplementary material file. DOI: 10.1107/S1600536812022210/hb6782Isup3.cml

Additional supplementary materials:  crystallographic information; 3D view; checkCIF report

## Figures and Tables

**Table 1:** Hydrogen-bond geometry (Å, °)

*D*—H⋯*A*	*D*—H	H⋯*A*	*D*⋯*A*	*D*—H⋯*A*
C22*B*—H22*D*⋯O2*A*	0.98	2.43	3.250 (3)	141
C17*B*—H17*B*⋯N1*B*^i^	0.95	2.62	3.524 (3)	159
C22*A*—H22*A*⋯O2*B*^ii^	0.98	2.43	3.250 (3)	141

Symmetry codes: (i) 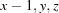; (ii) 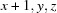.

**Fig. 1.**
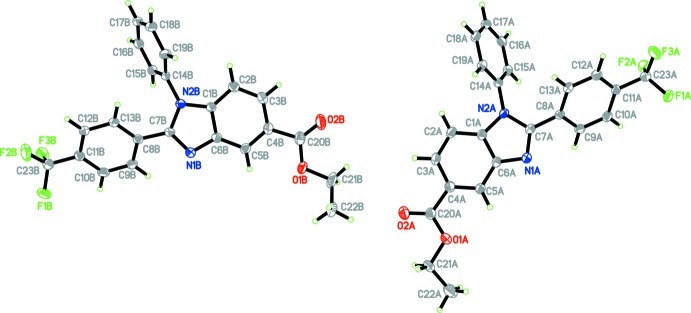
The molecular structure of the title compound, showing 50% probability displacement ellipsoids.

**Fig. 2.**
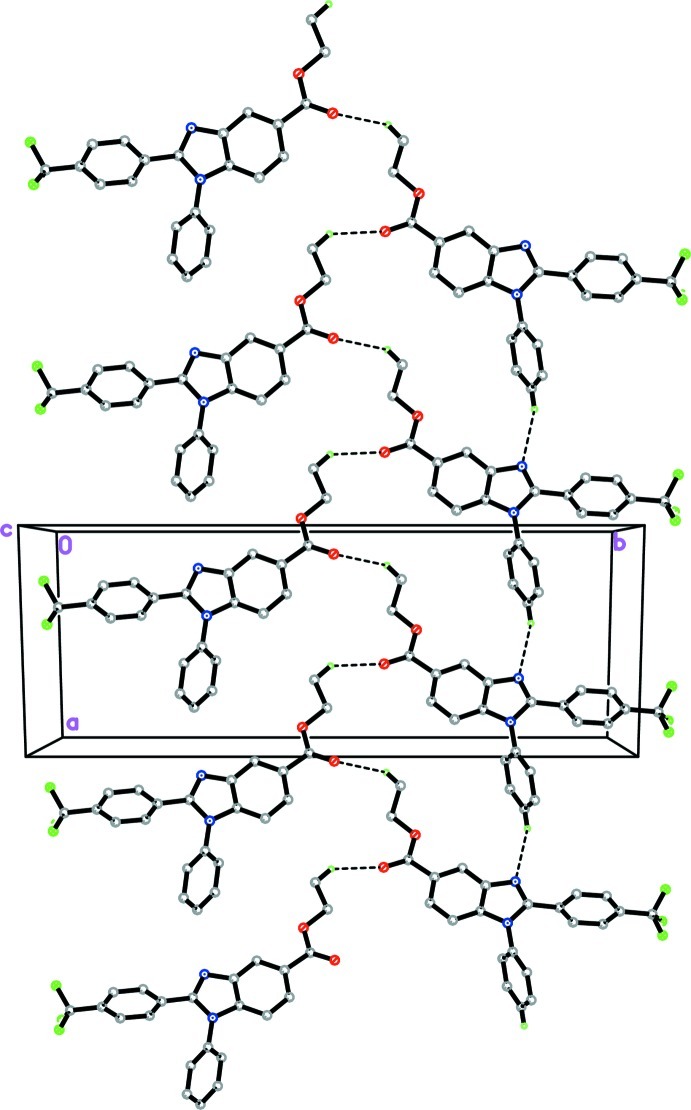
The crystal packing of the title compound. The H atoms not involved in the intermolecular interactions (dashed lines) have been omitted for clarity.

## supplementary crystallographic information

### Comment

The benzimidazole nucleus is an important pharmacophore in drug discovery (Spasov *et al.*, 1999). They thus generate a lot of pharmacological interests (Grassmann *et al.*, 2002; Demirayak *et al.*, 2002; Evans *et al.*, 1997). As part of our studies in this area, the crystal structure determination of the title compound was carried out and the results are presented here.

The asymmetric unit of the title compound (Fig 1), consists of two crystallographically independent molecules *A* and *B*. The benzimidazole ring system in both molecules, N1A/N2A/C1A—C7A and N1B/N2B/C1B—C7B, are essentially planar with a maximum deviation of 0.014 (2) Å at atom C7A and of 0.006 (2) Å at atom N2B, respectively. In molecule *A*, the benzimidazole ring (N1A/N2A/C1A—C7A) makes dihedral angles of 52.05 (8) and 33.70 (8)°, respectively with the phenyl ring (C14A–C19A) and the triflouromethyl-substituted phenyl ring (C8A–C13A). The corresponding dihedral angles in molecule *B* are 58.40 (8) and 25.90 (8)°.

In addition, the phenyl ring and the triflouromethyl-substituted phenyl ring form dihedral angles of 58.24 (10) and 60.83 (10)° in molecule *A* and *B*, respectively. Bond lengths and angles are within normal ranges and are comparable to related structure (Yoon *et al.*, 2011; Kassim *et al.*, 2012).

In the crystal (Fig. 2), C22B—H22D···O2A, C17B—H17B···N1B and C22A—H22A···O2B (Table 1) hydrogen bonds link the molecules into one-dimensional chains along *a*-axis. π–π interactions of *Cg*1···*Cg*2 = 3.6700 (12) Å (symmetry code: 1 - *x*,1 - *y*,2 - *z*) further stabilized the structure [*Cg*1 and *Cg*2 are the centroids of the N1A/N2A/C1A/C7A/C8A and C1B–C6B rings, respectively].

### Experimental

Ethyl 3-amino-4-(phenyl amino) benzoate (0.84 mmol) and sodium metabisulfite adduct of trifluoromethyl benzaldehyde (1.68 mmol) were dissolved in DMF. The reaction mixture was reflux at 130 °C for 2 h. After completion, the reaction mixture was diluted in ethyl acetate (20 ml) and washed with water (20 ml). The organic layer was collected, dried over Na_2_SO_4_ and the evaporated *in vacuo* to yield the product. The product was recrystallized from ethyl acetate as colourless blocks.

### Refinement

All H atoms were positioned geometrically [C–H = 0.95–0.99 Å] and refined using a riding model with *U*_iso_(H) = 1.2 and 1.5 *U*_eq_(C). A rotating group model was applied to the methyl groups. In the final refinement, two outliers (0 2 0 and 0 0 2) were omitted.

### Crystal data

**Table:**

C_23_H_17_F_3_N_2_O_2_	*F*(000) = 1696
*M**_r_* = 410.39	*D*_x_ = 1.437 Mg m^−^^3^
Monoclinic, *P*2_1_/*c*	Mo *K*α radiation, λ = 0.71073 Å
Hall symbol: -P 2ybc	Cell parameters from 7502 reflections
*a* = 9.8548 (2) Å	θ = 2.2–29.0°
*b* = 25.0714 (6) Å	µ = 0.11 mm^−^^1^
*c* = 16.0566 (4) Å	*T* = 100 K
β = 107.023 (1)°	Block, colourless
*V* = 3793.35 (15) Å^3^	0.43 × 0.37 × 0.28 mm
*Z* = 8	

### Data collection

**Table:**

Bruker SMART APEXII CCD diffractometer	10921 independent reflections
Radiation source: fine-focus sealed tube	5999 reflections with *I* > 2σ(*I*)
Graphite monochromator	*R*_int_ = 0.052
φ and ω scans	θ_max_ = 30.0°, θ_min_ = 2.1°
Absorption correction: multi-scan (*SADABS*; Bruker, 2009)	*h* = −13→8
*T*_min_ = 0.953, *T*_max_ = 0.969	*k* = −35→28
36855 measured reflections	*l* = −17→22

### Refinement

**Table:**

Refinement on *F*^2^	Primary atom site location: structure-invariant direct methods
Least-squares matrix: full	Secondary atom site location: difference Fourier map
*R*[*F*^2^ > 2σ(*F*^2^)] = 0.062	Hydrogen site location: inferred from neighbouring sites
*wR*(*F*^2^) = 0.142	H-atom parameters constrained
*S* = 1.03	*w* = 1/[σ^2^(*F*_o_^2^) + (0.0497*P*)^2^ + 1.1602*P*] where *P* = (*F*_o_^2^ + 2*F*_c_^2^)/3
10921 reflections	(Δ/σ)_max_ = 0.001
543 parameters	Δρ_max_ = 0.30 e Å^−^^3^
0 restraints	Δρ_min_ = −0.35 e Å^−^^3^

### Special details

**Table:**

Experimental. The crystal was placed in the cold stream of an Oxford Cryosystems Cobra open-flow nitrogen cryostat (Cosier & Glazer, 1986) operating at 100.0 (1) K.
Geometry. All e.s.d.'s (except the e.s.d. in the dihedral angle between two l.s. planes) are estimated using the full covariance matrix. The cell e.s.d.'s are taken into account individually in the estimation of e.s.d.'s in distances, angles and torsion angles; correlations between e.s.d.'s in cell parameters are only used when they are defined by crystal symmetry. An approximate (isotropic) treatment of cell e.s.d.'s is used for estimating e.s.d.'s involving l.s. planes.
Refinement. Refinement of *F*^2^ against ALL reflections. The weighted *R*-factor *wR* and goodness of fit *S* are based on *F*^2^, conventional *R*-factors *R* are based on *F*, with *F* set to zero for negative *F*^2^. The threshold expression of *F*^2^ > σ(*F*^2^) is used only for calculating *R*-factors(gt) *etc*. and is not relevant to the choice of reflections for refinement. *R*-factors based on *F*^2^ are statistically about twice as large as those based on *F*, and *R*- factors based on ALL data will be even larger.

### Fractional atomic coordinates and isotropic or equivalent isotropic displacement parameters (Å^2^)

**Table:**

	*x*	*y*	*z*	*U*_iso_*/*U*_eq_	
F1A	0.76297 (13)	1.01889 (5)	0.89337 (9)	0.0404 (4)	
F2A	0.55907 (13)	1.00949 (5)	0.79924 (8)	0.0359 (3)	
F3A	0.57601 (14)	1.01271 (5)	0.93564 (9)	0.0386 (3)	
O1A	1.05923 (15)	0.55832 (5)	0.88980 (10)	0.0290 (4)	
O2A	0.89188 (16)	0.50085 (6)	0.90308 (10)	0.0341 (4)	
N1A	0.82356 (17)	0.74226 (6)	0.88432 (11)	0.0218 (4)	
N2A	0.59828 (16)	0.72851 (6)	0.88711 (11)	0.0206 (4)	
C1A	0.6608 (2)	0.67850 (8)	0.89085 (13)	0.0212 (4)	
C2A	0.6093 (2)	0.62721 (8)	0.89629 (13)	0.0238 (5)	
H2AA	0.5148	0.6212	0.8976	0.029*	
C3A	0.7023 (2)	0.58550 (8)	0.89961 (13)	0.0250 (5)	
H3AA	0.6711	0.5500	0.9041	0.030*	
C4A	0.8416 (2)	0.59399 (8)	0.89653 (14)	0.0242 (5)	
C5A	0.8915 (2)	0.64522 (8)	0.89047 (13)	0.0227 (5)	
H5AA	0.9856	0.6510	0.8882	0.027*	
C6A	0.7995 (2)	0.68802 (8)	0.88787 (13)	0.0212 (4)	
C7A	0.7031 (2)	0.76499 (8)	0.88416 (13)	0.0211 (4)	
C8A	0.6837 (2)	0.82335 (8)	0.88447 (13)	0.0195 (4)	
C9A	0.7566 (2)	0.85508 (8)	0.84015 (14)	0.0229 (5)	
H9AA	0.8167	0.8389	0.8107	0.028*	
C10A	0.7417 (2)	0.91004 (8)	0.83904 (14)	0.0239 (5)	
H10A	0.7900	0.9314	0.8079	0.029*	
C11A	0.6559 (2)	0.93399 (8)	0.88337 (14)	0.0218 (4)	
C12A	0.5862 (2)	0.90302 (8)	0.92964 (13)	0.0240 (5)	
H12A	0.5296	0.9195	0.9612	0.029*	
C13A	0.5996 (2)	0.84796 (8)	0.92967 (13)	0.0220 (5)	
H13A	0.5509	0.8268	0.9608	0.026*	
C14A	0.4494 (2)	0.73733 (8)	0.87188 (13)	0.0198 (4)	
C15A	0.3736 (2)	0.76759 (8)	0.80114 (13)	0.0226 (5)	
H15A	0.4203	0.7830	0.7630	0.027*	
C16A	0.2291 (2)	0.77507 (8)	0.78670 (14)	0.0250 (5)	
H16A	0.1770	0.7961	0.7387	0.030*	
C17A	0.1603 (2)	0.75224 (8)	0.84142 (15)	0.0265 (5)	
H17A	0.0613	0.7575	0.8312	0.032*	
C18A	0.2361 (2)	0.72168 (8)	0.91109 (14)	0.0256 (5)	
H18A	0.1886	0.7055	0.9482	0.031*	
C19A	0.3810 (2)	0.71448 (8)	0.92729 (14)	0.0234 (5)	
H19A	0.4330	0.6940	0.9760	0.028*	
C20A	0.9312 (2)	0.54611 (8)	0.89753 (14)	0.0260 (5)	
C21A	1.1536 (2)	0.51374 (8)	0.89029 (16)	0.0330 (6)	
H21A	1.1126	0.4903	0.8396	0.040*	
H21B	1.1679	0.4925	0.9441	0.040*	
C22A	1.2901 (3)	0.53614 (10)	0.8861 (2)	0.0666 (10)	
H22A	1.3572	0.5071	0.8875	0.100*	
H22B	1.3287	0.5598	0.9360	0.100*	
H22C	1.2749	0.5564	0.8319	0.100*	
C23A	0.6388 (2)	0.99321 (8)	0.87871 (15)	0.0263 (5)	
F1B	0.26113 (14)	−0.10576 (5)	0.84724 (12)	0.0597 (5)	
F2B	0.09026 (16)	−0.10489 (5)	0.90442 (10)	0.0471 (4)	
F3B	0.04605 (15)	−0.09778 (5)	0.76703 (9)	0.0454 (4)	
O1B	0.53146 (16)	0.35301 (6)	0.86297 (12)	0.0435 (5)	
O2B	0.36752 (18)	0.41052 (6)	0.87921 (12)	0.0436 (4)	
N1B	0.29935 (17)	0.16935 (6)	0.85506 (11)	0.0221 (4)	
N2B	0.08626 (16)	0.18163 (6)	0.87819 (11)	0.0199 (4)	
C1B	0.1461 (2)	0.23184 (8)	0.88029 (13)	0.0200 (4)	
C2B	0.0975 (2)	0.28262 (8)	0.89265 (13)	0.0232 (5)	
H2BA	0.0080	0.2878	0.9026	0.028*	
C3B	0.1855 (2)	0.32487 (8)	0.88979 (13)	0.0248 (5)	
H3BA	0.1562	0.3600	0.8982	0.030*	
C4B	0.3183 (2)	0.31702 (8)	0.87455 (13)	0.0233 (5)	
C5B	0.3658 (2)	0.26639 (8)	0.86268 (13)	0.0228 (5)	
H5BA	0.4556	0.2612	0.8532	0.027*	
C6B	0.2778 (2)	0.22339 (8)	0.86507 (13)	0.0213 (4)	
C7B	0.1845 (2)	0.14569 (8)	0.86316 (13)	0.0200 (4)	
C8B	0.1679 (2)	0.08739 (8)	0.86005 (13)	0.0200 (4)	
C9B	0.2440 (2)	0.05866 (8)	0.81417 (14)	0.0239 (5)	
H9BA	0.3029	0.0771	0.7862	0.029*	
C10B	0.2346 (2)	0.00394 (8)	0.80904 (14)	0.0265 (5)	
H10B	0.2856	−0.0152	0.7768	0.032*	
C11B	0.1506 (2)	−0.02321 (8)	0.85099 (14)	0.0245 (5)	
C12B	0.0767 (2)	0.00470 (8)	0.89859 (14)	0.0242 (5)	
H12B	0.0205	−0.0139	0.9281	0.029*	
C13B	0.0855 (2)	0.05993 (8)	0.90287 (14)	0.0228 (5)	
H13B	0.0348	0.0791	0.9353	0.027*	
C14B	−0.0593 (2)	0.17171 (7)	0.87440 (13)	0.0199 (4)	
C15B	−0.1489 (2)	0.14730 (8)	0.80140 (14)	0.0226 (5)	
H15B	−0.1146	0.1368	0.7544	0.027*	
C16B	−0.2889 (2)	0.13850 (8)	0.79805 (15)	0.0257 (5)	
H16B	−0.3506	0.1213	0.7487	0.031*	
C17B	−0.3405 (2)	0.15442 (8)	0.86555 (15)	0.0281 (5)	
H17B	−0.4370	0.1484	0.8626	0.034*	
C18B	−0.2501 (2)	0.17911 (8)	0.93713 (16)	0.0303 (5)	
H18B	−0.2852	0.1904	0.9834	0.036*	
C19B	−0.1085 (2)	0.18781 (8)	0.94274 (14)	0.0258 (5)	
H19B	−0.0466	0.2045	0.9926	0.031*	
C20B	0.4053 (2)	0.36554 (9)	0.87282 (14)	0.0284 (5)	
C21B	0.6254 (3)	0.39661 (10)	0.8563 (2)	0.0538 (8)	
H21C	0.6017	0.4291	0.8842	0.065*	
H21D	0.6146	0.4047	0.7943	0.065*	
C22B	0.7715 (3)	0.37996 (10)	0.90028 (18)	0.0447 (7)	
H22D	0.8378	0.4062	0.8891	0.067*	
H22E	0.7892	0.3451	0.8781	0.067*	
H22F	0.7851	0.3775	0.9631	0.067*	
C23B	0.1376 (2)	−0.08240 (9)	0.84303 (16)	0.0308 (5)	

### Atomic displacement parameters (Å^2^)

**Table:**

	*U*^11^	*U*^22^	*U*^33^	*U*^12^	*U*^13^	*U*^23^
F1A	0.0245 (7)	0.0212 (7)	0.0727 (11)	−0.0050 (5)	0.0097 (7)	−0.0006 (7)
F2A	0.0357 (8)	0.0293 (7)	0.0399 (8)	0.0048 (6)	0.0065 (6)	0.0090 (6)
F3A	0.0476 (9)	0.0257 (7)	0.0482 (9)	0.0051 (6)	0.0230 (7)	−0.0037 (6)
O1A	0.0285 (8)	0.0202 (7)	0.0400 (10)	0.0043 (6)	0.0125 (7)	0.0010 (7)
O2A	0.0340 (9)	0.0221 (8)	0.0438 (10)	−0.0001 (7)	0.0073 (8)	0.0012 (7)
N1A	0.0199 (9)	0.0203 (9)	0.0249 (10)	−0.0020 (7)	0.0059 (7)	−0.0009 (7)
N2A	0.0154 (8)	0.0194 (9)	0.0263 (10)	−0.0015 (7)	0.0051 (7)	0.0011 (7)
C1A	0.0211 (11)	0.0207 (10)	0.0200 (11)	−0.0026 (8)	0.0031 (8)	−0.0006 (9)
C2A	0.0190 (11)	0.0244 (11)	0.0263 (12)	−0.0040 (8)	0.0039 (9)	0.0015 (9)
C3A	0.0252 (11)	0.0204 (11)	0.0257 (12)	−0.0052 (9)	0.0015 (9)	0.0021 (9)
C4A	0.0230 (11)	0.0225 (11)	0.0245 (12)	0.0001 (9)	0.0030 (9)	0.0002 (9)
C5A	0.0210 (11)	0.0215 (11)	0.0246 (12)	−0.0020 (9)	0.0049 (9)	−0.0005 (9)
C6A	0.0198 (10)	0.0210 (10)	0.0213 (12)	−0.0036 (8)	0.0038 (8)	−0.0025 (9)
C7A	0.0175 (10)	0.0246 (11)	0.0203 (11)	−0.0035 (8)	0.0043 (8)	0.0001 (9)
C8A	0.0154 (10)	0.0219 (10)	0.0202 (11)	−0.0007 (8)	0.0036 (8)	−0.0023 (9)
C9A	0.0185 (10)	0.0257 (11)	0.0252 (12)	−0.0004 (8)	0.0075 (9)	−0.0009 (9)
C10A	0.0229 (11)	0.0223 (11)	0.0271 (12)	−0.0040 (9)	0.0082 (9)	0.0018 (9)
C11A	0.0185 (10)	0.0188 (10)	0.0254 (12)	−0.0016 (8)	0.0020 (9)	−0.0010 (9)
C12A	0.0199 (11)	0.0268 (11)	0.0249 (12)	−0.0002 (9)	0.0062 (9)	−0.0046 (9)
C13A	0.0193 (11)	0.0233 (11)	0.0233 (12)	−0.0042 (8)	0.0058 (9)	0.0005 (9)
C14A	0.0158 (10)	0.0197 (10)	0.0227 (11)	−0.0023 (8)	0.0039 (8)	−0.0036 (9)
C15A	0.0244 (11)	0.0205 (10)	0.0227 (12)	−0.0020 (8)	0.0064 (9)	−0.0021 (9)
C16A	0.0224 (11)	0.0200 (10)	0.0293 (12)	0.0022 (8)	0.0023 (9)	−0.0029 (9)
C17A	0.0193 (11)	0.0247 (11)	0.0359 (14)	−0.0019 (9)	0.0084 (10)	−0.0101 (10)
C18A	0.0234 (11)	0.0249 (11)	0.0308 (13)	−0.0076 (9)	0.0116 (10)	−0.0051 (10)
C19A	0.0226 (11)	0.0232 (11)	0.0231 (12)	−0.0051 (9)	0.0050 (9)	−0.0021 (9)
C20A	0.0252 (12)	0.0230 (12)	0.0268 (13)	−0.0010 (9)	0.0027 (9)	0.0011 (9)
C21A	0.0357 (13)	0.0208 (11)	0.0447 (15)	0.0066 (10)	0.0154 (11)	0.0002 (10)
C22A	0.061 (2)	0.0321 (15)	0.128 (3)	0.0102 (13)	0.062 (2)	0.0098 (17)
C23A	0.0204 (11)	0.0253 (11)	0.0330 (14)	0.0005 (9)	0.0072 (10)	−0.0014 (10)
F1B	0.0283 (8)	0.0248 (7)	0.1254 (15)	0.0052 (6)	0.0214 (9)	−0.0106 (8)
F2B	0.0639 (10)	0.0245 (7)	0.0554 (10)	−0.0010 (7)	0.0214 (8)	0.0070 (7)
F3B	0.0501 (9)	0.0302 (7)	0.0482 (9)	−0.0089 (6)	0.0025 (7)	−0.0122 (7)
O1B	0.0266 (9)	0.0250 (8)	0.0791 (14)	−0.0038 (7)	0.0159 (9)	0.0166 (9)
O2B	0.0593 (12)	0.0212 (9)	0.0592 (12)	−0.0064 (8)	0.0310 (10)	−0.0041 (8)
N1B	0.0210 (9)	0.0203 (9)	0.0251 (10)	0.0012 (7)	0.0070 (8)	0.0017 (7)
N2B	0.0164 (8)	0.0196 (9)	0.0235 (10)	0.0008 (7)	0.0056 (7)	−0.0013 (7)
C1B	0.0182 (10)	0.0203 (10)	0.0197 (11)	0.0001 (8)	0.0030 (8)	−0.0003 (9)
C2B	0.0202 (11)	0.0244 (11)	0.0226 (12)	0.0016 (9)	0.0027 (9)	−0.0007 (9)
C3B	0.0293 (12)	0.0185 (10)	0.0231 (12)	0.0021 (9)	0.0021 (9)	−0.0009 (9)
C4B	0.0256 (11)	0.0220 (11)	0.0195 (11)	−0.0035 (9)	0.0023 (9)	0.0016 (9)
C5B	0.0209 (11)	0.0240 (11)	0.0233 (12)	−0.0008 (9)	0.0062 (9)	0.0040 (9)
C6B	0.0210 (11)	0.0219 (11)	0.0205 (11)	0.0024 (8)	0.0052 (9)	0.0011 (9)
C7B	0.0168 (10)	0.0233 (11)	0.0198 (11)	0.0019 (8)	0.0053 (8)	0.0004 (9)
C8B	0.0160 (10)	0.0208 (10)	0.0213 (11)	0.0011 (8)	0.0025 (8)	−0.0009 (9)
C9B	0.0204 (11)	0.0254 (11)	0.0270 (12)	−0.0006 (9)	0.0085 (9)	−0.0021 (9)
C10B	0.0212 (11)	0.0276 (12)	0.0320 (13)	0.0026 (9)	0.0099 (10)	−0.0052 (10)
C11B	0.0176 (10)	0.0215 (11)	0.0299 (13)	0.0009 (8)	0.0000 (9)	−0.0009 (9)
C12B	0.0197 (11)	0.0225 (11)	0.0310 (13)	0.0007 (9)	0.0081 (9)	0.0031 (10)
C13B	0.0203 (11)	0.0218 (11)	0.0275 (12)	0.0019 (8)	0.0089 (9)	−0.0011 (9)
C14B	0.0148 (10)	0.0168 (10)	0.0277 (12)	0.0003 (8)	0.0056 (9)	0.0011 (9)
C15B	0.0230 (11)	0.0200 (10)	0.0239 (12)	0.0010 (8)	0.0053 (9)	0.0012 (9)
C16B	0.0191 (11)	0.0203 (11)	0.0330 (13)	0.0006 (8)	0.0004 (9)	0.0001 (9)
C17B	0.0167 (10)	0.0218 (11)	0.0458 (15)	0.0005 (9)	0.0090 (10)	−0.0009 (10)
C18B	0.0268 (12)	0.0275 (12)	0.0415 (15)	0.0017 (9)	0.0176 (11)	−0.0053 (11)
C19B	0.0208 (11)	0.0271 (11)	0.0299 (13)	−0.0008 (9)	0.0081 (9)	−0.0055 (10)
C20B	0.0344 (13)	0.0253 (12)	0.0227 (12)	−0.0038 (10)	0.0040 (10)	0.0037 (10)
C21B	0.0387 (16)	0.0342 (14)	0.086 (2)	−0.0119 (12)	0.0140 (15)	0.0244 (15)
C22B	0.0409 (16)	0.0299 (13)	0.0648 (19)	−0.0133 (11)	0.0179 (14)	−0.0055 (13)
C23B	0.0233 (12)	0.0237 (11)	0.0446 (16)	−0.0015 (9)	0.0090 (11)	−0.0025 (11)

### Geometric parameters (Å, º)

**Table:**

F1A—C23A	1.341 (2)	F1B—C23B	1.335 (2)
F2A—C23A	1.351 (2)	F2B—C23B	1.333 (3)
F3A—C23A	1.337 (2)	F3B—C23B	1.344 (3)
O1A—C20A	1.339 (2)	O1B—C20B	1.336 (3)
O1A—C21A	1.453 (2)	O1B—C21B	1.457 (3)
O2A—C20A	1.211 (2)	O2B—C20B	1.201 (3)
N1A—C7A	1.316 (2)	N1B—C7B	1.317 (2)
N1A—C6A	1.385 (2)	N1B—C6B	1.388 (2)
N2A—C1A	1.390 (2)	N2B—C1B	1.386 (2)
N2A—C7A	1.391 (2)	N2B—C7B	1.394 (2)
N2A—C14A	1.432 (2)	N2B—C14B	1.440 (2)
C1A—C2A	1.394 (3)	C1B—C2B	1.395 (3)
C1A—C6A	1.402 (3)	C1B—C6B	1.405 (3)
C2A—C3A	1.381 (3)	C2B—C3B	1.378 (3)
C2A—H2AA	0.9500	C2B—H2BA	0.9500
C3A—C4A	1.404 (3)	C3B—C4B	1.414 (3)
C3A—H3AA	0.9500	C3B—H3BA	0.9500
C4A—C5A	1.389 (3)	C4B—C5B	1.385 (3)
C4A—C20A	1.488 (3)	C4B—C20B	1.493 (3)
C5A—C6A	1.397 (3)	C5B—C6B	1.392 (3)
C5A—H5AA	0.9500	C5B—H5BA	0.9500
C7A—C8A	1.476 (3)	C7B—C8B	1.470 (3)
C8A—C13A	1.395 (3)	C8B—C13B	1.390 (3)
C8A—C9A	1.399 (3)	C8B—C9B	1.396 (3)
C9A—C10A	1.385 (3)	C9B—C10B	1.376 (3)
C9A—H9AA	0.9500	C9B—H9BA	0.9500
C10A—C11A	1.390 (3)	C10B—C11B	1.389 (3)
C10A—H10A	0.9500	C10B—H10B	0.9500
C11A—C12A	1.388 (3)	C11B—C12B	1.389 (3)
C11A—C23A	1.494 (3)	C11B—C23B	1.492 (3)
C12A—C13A	1.387 (3)	C12B—C13B	1.388 (3)
C12A—H12A	0.9500	C12B—H12B	0.9500
C13A—H13A	0.9500	C13B—H13B	0.9500
C14A—C19A	1.388 (3)	C14B—C19B	1.383 (3)
C14A—C15A	1.388 (3)	C14B—C15B	1.386 (3)
C15A—C16A	1.387 (3)	C15B—C16B	1.382 (3)
C15A—H15A	0.9500	C15B—H15B	0.9500
C16A—C17A	1.381 (3)	C16B—C17B	1.384 (3)
C16A—H16A	0.9500	C16B—H16B	0.9500
C17A—C18A	1.381 (3)	C17B—C18B	1.378 (3)
C17A—H17A	0.9500	C17B—H17B	0.9500
C18A—C19A	1.386 (3)	C18B—C19B	1.388 (3)
C18A—H18A	0.9500	C18B—H18B	0.9500
C19A—H19A	0.9500	C19B—H19B	0.9500
C21A—C22A	1.477 (3)	C21B—C22B	1.466 (4)
C21A—H21A	0.9900	C21B—H21C	0.9900
C21A—H21B	0.9900	C21B—H21D	0.9900
C22A—H22A	0.9800	C22B—H22D	0.9800
C22A—H22B	0.9800	C22B—H22E	0.9800
C22A—H22C	0.9800	C22B—H22F	0.9800
			
C20A—O1A—C21A	116.27 (16)	C20B—O1B—C21B	117.78 (18)
C7A—N1A—C6A	105.06 (16)	C7B—N1B—C6B	105.23 (16)
C1A—N2A—C7A	105.71 (15)	C1B—N2B—C7B	106.20 (15)
C1A—N2A—C14A	124.42 (16)	C1B—N2B—C14B	124.70 (16)
C7A—N2A—C14A	128.78 (16)	C7B—N2B—C14B	127.95 (16)
N2A—C1A—C2A	132.04 (18)	N2B—C1B—C2B	132.15 (18)
N2A—C1A—C6A	105.60 (16)	N2B—C1B—C6B	105.46 (16)
C2A—C1A—C6A	122.36 (18)	C2B—C1B—C6B	122.38 (18)
C3A—C2A—C1A	116.82 (19)	C3B—C2B—C1B	116.82 (19)
C3A—C2A—H2AA	121.6	C3B—C2B—H2BA	121.6
C1A—C2A—H2AA	121.6	C1B—C2B—H2BA	121.6
C2A—C3A—C4A	121.89 (19)	C2B—C3B—C4B	121.48 (19)
C2A—C3A—H3AA	119.1	C2B—C3B—H3BA	119.3
C4A—C3A—H3AA	119.1	C4B—C3B—H3BA	119.3
C5A—C4A—C3A	120.80 (19)	C5B—C4B—C3B	121.23 (18)
C5A—C4A—C20A	121.74 (19)	C5B—C4B—C20B	121.66 (19)
C3A—C4A—C20A	117.43 (18)	C3B—C4B—C20B	117.10 (19)
C4A—C5A—C6A	118.21 (19)	C4B—C5B—C6B	117.87 (19)
C4A—C5A—H5AA	120.9	C4B—C5B—H5BA	121.1
C6A—C5A—H5AA	120.9	C6B—C5B—H5BA	121.1
N1A—C6A—C5A	129.64 (18)	N1B—C6B—C5B	129.42 (18)
N1A—C6A—C1A	110.44 (17)	N1B—C6B—C1B	110.38 (17)
C5A—C6A—C1A	119.91 (18)	C5B—C6B—C1B	120.20 (18)
N1A—C7A—N2A	113.17 (17)	N1B—C7B—N2B	112.72 (17)
N1A—C7A—C8A	123.14 (17)	N1B—C7B—C8B	122.31 (17)
N2A—C7A—C8A	123.64 (17)	N2B—C7B—C8B	124.91 (17)
C13A—C8A—C9A	119.04 (18)	C13B—C8B—C9B	119.18 (18)
C13A—C8A—C7A	122.50 (18)	C13B—C8B—C7B	123.42 (18)
C9A—C8A—C7A	118.43 (17)	C9B—C8B—C7B	117.35 (18)
C10A—C9A—C8A	120.25 (19)	C10B—C9B—C8B	120.60 (19)
C10A—C9A—H9AA	119.9	C10B—C9B—H9BA	119.7
C8A—C9A—H9AA	119.9	C8B—C9B—H9BA	119.7
C9A—C10A—C11A	120.08 (19)	C9B—C10B—C11B	119.93 (19)
C9A—C10A—H10A	120.0	C9B—C10B—H10B	120.0
C11A—C10A—H10A	120.0	C11B—C10B—H10B	120.0
C12A—C11A—C10A	120.19 (18)	C10B—C11B—C12B	120.19 (19)
C12A—C11A—C23A	121.10 (19)	C10B—C11B—C23B	119.67 (19)
C10A—C11A—C23A	118.71 (18)	C12B—C11B—C23B	120.13 (19)
C13A—C12A—C11A	119.70 (19)	C13B—C12B—C11B	119.65 (19)
C13A—C12A—H12A	120.2	C13B—C12B—H12B	120.2
C11A—C12A—H12A	120.2	C11B—C12B—H12B	120.2
C12A—C13A—C8A	120.71 (19)	C12B—C13B—C8B	120.42 (19)
C12A—C13A—H13A	119.6	C12B—C13B—H13B	119.8
C8A—C13A—H13A	119.6	C8B—C13B—H13B	119.8
C19A—C14A—C15A	120.24 (18)	C19B—C14B—C15B	121.01 (19)
C19A—C14A—N2A	119.52 (18)	C19B—C14B—N2B	119.50 (18)
C15A—C14A—N2A	120.22 (18)	C15B—C14B—N2B	119.47 (18)
C16A—C15A—C14A	119.41 (19)	C16B—C15B—C14B	119.0 (2)
C16A—C15A—H15A	120.3	C16B—C15B—H15B	120.5
C14A—C15A—H15A	120.3	C14B—C15B—H15B	120.5
C17A—C16A—C15A	120.6 (2)	C15B—C16B—C17B	121.0 (2)
C17A—C16A—H16A	119.7	C15B—C16B—H16B	119.5
C15A—C16A—H16A	119.7	C17B—C16B—H16B	119.5
C18A—C17A—C16A	119.7 (2)	C18B—C17B—C16B	119.1 (2)
C18A—C17A—H17A	120.2	C18B—C17B—H17B	120.4
C16A—C17A—H17A	120.2	C16B—C17B—H17B	120.4
C17A—C18A—C19A	120.5 (2)	C17B—C18B—C19B	121.1 (2)
C17A—C18A—H18A	119.7	C17B—C18B—H18B	119.4
C19A—C18A—H18A	119.7	C19B—C18B—H18B	119.4
C18A—C19A—C14A	119.6 (2)	C14B—C19B—C18B	118.8 (2)
C18A—C19A—H19A	120.2	C14B—C19B—H19B	120.6
C14A—C19A—H19A	120.2	C18B—C19B—H19B	120.6
O2A—C20A—O1A	123.51 (19)	O2B—C20B—O1B	123.6 (2)
O2A—C20A—C4A	123.71 (19)	O2B—C20B—C4B	124.7 (2)
O1A—C20A—C4A	112.77 (17)	O1B—C20B—C4B	111.72 (18)
O1A—C21A—C22A	107.29 (18)	O1B—C21B—C22B	107.8 (2)
O1A—C21A—H21A	110.3	O1B—C21B—H21C	110.1
C22A—C21A—H21A	110.3	C22B—C21B—H21C	110.1
O1A—C21A—H21B	110.3	O1B—C21B—H21D	110.1
C22A—C21A—H21B	110.3	C22B—C21B—H21D	110.1
H21A—C21A—H21B	108.5	H21C—C21B—H21D	108.5
C21A—C22A—H22A	109.5	C21B—C22B—H22D	109.5
C21A—C22A—H22B	109.5	C21B—C22B—H22E	109.5
H22A—C22A—H22B	109.5	H22D—C22B—H22E	109.5
C21A—C22A—H22C	109.5	C21B—C22B—H22F	109.5
H22A—C22A—H22C	109.5	H22D—C22B—H22F	109.5
H22B—C22A—H22C	109.5	H22E—C22B—H22F	109.5
F3A—C23A—F1A	106.59 (17)	F2B—C23B—F1B	106.76 (19)
F3A—C23A—F2A	106.13 (16)	F2B—C23B—F3B	105.26 (17)
F1A—C23A—F2A	105.61 (17)	F1B—C23B—F3B	106.24 (18)
F3A—C23A—C11A	113.40 (18)	F2B—C23B—C11B	113.44 (19)
F1A—C23A—C11A	112.59 (17)	F1B—C23B—C11B	112.17 (17)
F2A—C23A—C11A	111.94 (17)	F3B—C23B—C11B	112.42 (18)
			
C7A—N2A—C1A—C2A	178.8 (2)	C7B—N2B—C1B—C2B	179.8 (2)
C14A—N2A—C1A—C2A	−12.3 (3)	C14B—N2B—C1B—C2B	11.3 (3)
C7A—N2A—C1A—C6A	−1.3 (2)	C7B—N2B—C1B—C6B	0.8 (2)
C14A—N2A—C1A—C6A	167.61 (18)	C14B—N2B—C1B—C6B	−167.79 (18)
N2A—C1A—C2A—C3A	−179.5 (2)	N2B—C1B—C2B—C3B	−179.3 (2)
C6A—C1A—C2A—C3A	0.7 (3)	C6B—C1B—C2B—C3B	−0.3 (3)
C1A—C2A—C3A—C4A	−0.8 (3)	C1B—C2B—C3B—C4B	0.3 (3)
C2A—C3A—C4A—C5A	0.4 (3)	C2B—C3B—C4B—C5B	−0.6 (3)
C2A—C3A—C4A—C20A	−177.97 (19)	C2B—C3B—C4B—C20B	−179.96 (19)
C3A—C4A—C5A—C6A	0.2 (3)	C3B—C4B—C5B—C6B	0.8 (3)
C20A—C4A—C5A—C6A	178.52 (19)	C20B—C4B—C5B—C6B	−179.85 (19)
C7A—N1A—C6A—C5A	−179.2 (2)	C7B—N1B—C6B—C5B	179.8 (2)
C7A—N1A—C6A—C1A	−0.7 (2)	C7B—N1B—C6B—C1B	0.5 (2)
C4A—C5A—C6A—N1A	178.1 (2)	C4B—C5B—C6B—N1B	179.9 (2)
C4A—C5A—C6A—C1A	−0.4 (3)	C4B—C5B—C6B—C1B	−0.8 (3)
N2A—C1A—C6A—N1A	1.3 (2)	N2B—C1B—C6B—N1B	−0.8 (2)
C2A—C1A—C6A—N1A	−178.83 (18)	C2B—C1B—C6B—N1B	−179.96 (18)
N2A—C1A—C6A—C5A	−179.99 (18)	N2B—C1B—C6B—C5B	179.78 (18)
C2A—C1A—C6A—C5A	−0.1 (3)	C2B—C1B—C6B—C5B	0.6 (3)
C6A—N1A—C7A—N2A	−0.2 (2)	C6B—N1B—C7B—N2B	0.0 (2)
C6A—N1A—C7A—C8A	177.40 (18)	C6B—N1B—C7B—C8B	−177.55 (18)
C1A—N2A—C7A—N1A	1.0 (2)	C1B—N2B—C7B—N1B	−0.5 (2)
C14A—N2A—C7A—N1A	−167.28 (18)	C14B—N2B—C7B—N1B	167.55 (18)
C1A—N2A—C7A—C8A	−176.60 (18)	C1B—N2B—C7B—C8B	176.97 (18)
C14A—N2A—C7A—C8A	15.1 (3)	C14B—N2B—C7B—C8B	−15.0 (3)
N1A—C7A—C8A—C13A	−143.8 (2)	N1B—C7B—C8B—C13B	151.6 (2)
N2A—C7A—C8A—C13A	33.6 (3)	N2B—C7B—C8B—C13B	−25.7 (3)
N1A—C7A—C8A—C9A	34.0 (3)	N1B—C7B—C8B—C9B	−25.9 (3)
N2A—C7A—C8A—C9A	−148.65 (19)	N2B—C7B—C8B—C9B	156.83 (19)
C13A—C8A—C9A—C10A	−2.0 (3)	C13B—C8B—C9B—C10B	1.9 (3)
C7A—C8A—C9A—C10A	−179.83 (18)	C7B—C8B—C9B—C10B	179.48 (19)
C8A—C9A—C10A—C11A	1.2 (3)	C8B—C9B—C10B—C11B	−1.1 (3)
C9A—C10A—C11A—C12A	0.7 (3)	C9B—C10B—C11B—C12B	−0.4 (3)
C9A—C10A—C11A—C23A	−178.25 (19)	C9B—C10B—C11B—C23B	178.2 (2)
C10A—C11A—C12A—C13A	−1.6 (3)	C10B—C11B—C12B—C13B	1.0 (3)
C23A—C11A—C12A—C13A	177.27 (19)	C23B—C11B—C12B—C13B	−177.6 (2)
C11A—C12A—C13A—C8A	0.7 (3)	C11B—C12B—C13B—C8B	−0.2 (3)
C9A—C8A—C13A—C12A	1.1 (3)	C9B—C8B—C13B—C12B	−1.3 (3)
C7A—C8A—C13A—C12A	178.79 (18)	C7B—C8B—C13B—C12B	−178.71 (19)
C1A—N2A—C14A—C19A	57.5 (3)	C1B—N2B—C14B—C19B	−63.5 (3)
C7A—N2A—C14A—C19A	−136.2 (2)	C7B—N2B—C14B—C19B	130.5 (2)
C1A—N2A—C14A—C15A	−121.2 (2)	C1B—N2B—C14B—C15B	115.0 (2)
C7A—N2A—C14A—C15A	45.1 (3)	C7B—N2B—C14B—C15B	−51.0 (3)
C19A—C14A—C15A—C16A	0.4 (3)	C19B—C14B—C15B—C16B	−0.8 (3)
N2A—C14A—C15A—C16A	179.07 (17)	N2B—C14B—C15B—C16B	−179.30 (17)
C14A—C15A—C16A—C17A	−0.7 (3)	C14B—C15B—C16B—C17B	1.0 (3)
C15A—C16A—C17A—C18A	0.0 (3)	C15B—C16B—C17B—C18B	−0.4 (3)
C16A—C17A—C18A—C19A	1.1 (3)	C16B—C17B—C18B—C19B	−0.5 (3)
C17A—C18A—C19A—C14A	−1.3 (3)	C15B—C14B—C19B—C18B	0.0 (3)
C15A—C14A—C19A—C18A	0.6 (3)	N2B—C14B—C19B—C18B	178.46 (18)
N2A—C14A—C19A—C18A	−178.09 (17)	C17B—C18B—C19B—C14B	0.7 (3)
C21A—O1A—C20A—O2A	−1.4 (3)	C21B—O1B—C20B—O2B	−2.5 (3)
C21A—O1A—C20A—C4A	179.94 (18)	C21B—O1B—C20B—C4B	177.3 (2)
C5A—C4A—C20A—O2A	179.7 (2)	C5B—C4B—C20B—O2B	176.9 (2)
C3A—C4A—C20A—O2A	−2.0 (3)	C3B—C4B—C20B—O2B	−3.8 (3)
C5A—C4A—C20A—O1A	−1.6 (3)	C5B—C4B—C20B—O1B	−2.9 (3)
C3A—C4A—C20A—O1A	176.72 (18)	C3B—C4B—C20B—O1B	176.40 (18)
C20A—O1A—C21A—C22A	−176.9 (2)	C20B—O1B—C21B—C22B	144.2 (2)
C12A—C11A—C23A—F3A	12.5 (3)	C10B—C11B—C23B—F2B	161.60 (19)
C10A—C11A—C23A—F3A	−168.60 (18)	C12B—C11B—C23B—F2B	−19.8 (3)
C12A—C11A—C23A—F1A	133.6 (2)	C10B—C11B—C23B—F1B	40.5 (3)
C10A—C11A—C23A—F1A	−47.5 (3)	C12B—C11B—C23B—F1B	−140.8 (2)
C12A—C11A—C23A—F2A	−107.6 (2)	C10B—C11B—C23B—F3B	−79.1 (2)
C10A—C11A—C23A—F2A	71.4 (2)	C12B—C11B—C23B—F3B	99.5 (2)

### Hydrogen-bond geometry (Å, º)

**Table:**

*D*—H···*A*	*D*—H	H···*A*	*D*···*A*	*D*—H···*A*
C22*B*—H22*D*···O2*A*	0.98	2.43	3.250 (3)	141
C17*B*—H17*B*···N1*B*^i^	0.95	2.62	3.524 (3)	159
C22*A*—H22*A*···O2*B*^ii^	0.98	2.43	3.250 (3)	141

Symmetry codes: (i) *x*−1, *y*, *z*; (ii) *x*+1, *y*, *z*.
